# The effects of different electrolyte composition in dialysate on QTc interval; a controlled trial

**DOI:** 10.15171/jrip.2016.32

**Published:** 2016-04-06

**Authors:** Foroogh Sabzghabaei, Seyed Alireza Heydariezade, Rashin S Joodat

**Affiliations:** ^1^Department of Medicine, Firoozgar Hospital, Iran University of Medical Sciences, Tehran, Iran

**Keywords:** Hemodialysis, QT interval, Dialysate fluid, Cardiovascular disease

## Abstract

**Introduction:** Hemodialysis (HD) has impact on the cardiovascular system by inducing changes in the characteristics of body fluids such as PH, temperature and electrolyte concentrations. In the previous studies, prolongation of the QT interval and increase of QT dispersion have been reported during HD sessions. These changes were more significant while using solutions with less potassium and higher bicarbonate during dialysis.

**Objectives:** The aim of our study was to investigate the effects of different potassium and bicarbonate concentrations on electrocardiography (ECG) parameters and the electrochemical balance of cell membranes.

**Patients and Methods:** This is a double blind controlled clinical trial with crossover design. This interventional study has been conducted on 36 patients over 18 years who undergoing HD 3 times a week for at least 6 months. Twelve-lead ECG has been obtained before starting and one hour after end of each HD session. The QTc was measured and changes recorded by a cardiologist. Correlations were evaluated by univariate regression analysis.

**Results:** 54.38 years (16 to 77 years), 66.7% were male. No significant increase in QT interval has been seen while dialyzing with 2 meq/l potassium and 24 meq/l bicarbonate, 2 meq/l potassium and 28 meq/l bicarbonate and 3 meq/l potassium and 24 meq/l bicarbonate beside high calcium (2.5 meq/l) dialysate was conducted. Age, gender, serum calcium and serum bicarbonate level before HD session did not influence the mean QT intervals before and after dialysis.

**Conclusion:** Concentration of potassium beside moderate dose of bicarbonate in dialysis bath had not any significant influence on QT intervals after dialysis.

**Implication for health policy/practice/research/medical education:**

Different concentration of potassium beside moderate dose of bicarbonate in dialysis bath had not any significant influence on QT intervals after dialysis.

## Introduction

Risk of cardiovascular disease increases in end stage renal disease (ESRD) patients undergoing maintenance hemodialysis (HD). This increased risk is due to renal and extra-renal factors (1-3).

Moreover, in HD patients, high mortality rate is due to HD complications such as arrhythmia and sudden cardiac death during HD sessions (2,3).

HD influences the cardiovascular system by inducing changes in the characteristics of body fluids, such as PH, temperature and electrolyte concentrations (4).

Simple diffusion is the mechanism of removal of potassium during HD (5) and is determined by the blood–dialysate concentration gradient, which generates diffusive–convective flows through the HD membrane (4,5). The use of dialysis baths with low potassium content presents a high gradient between plasma and HD bath which can lead to decrease plasma potassium levels. This can cause neural and skeletal muscle dysfunction (6,7). Moreover, decreased potassium level in plasma can induce cardiac arrhythmias. Also, hypocalcemia is associated with an increase in action potential duration and QT interval prolongation (8). However, high concentration of potassium in the dialysis bath, decreases removal of potassium from plasma and makes HD less effective.

Electrocardiography (ECG) is used to detect arrhythmias such as SVPCs (supraventricular premature contractions) and PVCs (premature ventricular contractions), ST segment elevation, inverted T wave and atrial fibrillation (AF) which are reliable markers of the altered electric potential of the myocytes (9,10). As mentioned before, HD alters the duration and homogeneity of ventricular repolarization. The QT interval and the QT interval corrected for heart rate (QTc) reflect the duration of ventricular depolarization and repolarization. Previous studies have shown that QTc is very sensitive to alterations in plasma potassium concentration and PH (11,12). QT dispersion (QTd) and QTc dispersion (QTcd) (defined as the difference between the maximal and minimal QT and QTc, respectively) represent the variability of cardiac repolarization, and they are found to be significantly prolonged during HD and are considered to be important markers of the risk of severe, even fatal cardiac arrhythmias (11,12).

In the previous studies, prolongation of the QT interval, (13-15) an increase of QT dispersion, (16-18) and an alteration of the capability to adapt QT interval to heart rate changes (19) have been reported during HD sessions.

A conducted study on 22 HD patients in 2011, the influence of different concentrations of potassium (2 and 3 meq/l), calcium (1.25, 1.5 and 1.75 meq/l) and bicarbonate (30 and 34 meq/l) in dialysate on QTc interval were tested. Results have shown that dialysis bath with less potassium, less calcium and highest concentration of bicarbonate causes significant increase in QTc. They concluded that electrolyte and bicarbonate concentrations in dialysate are important determinants of the QT duration (20).

In a study by Genovesi et al in 2009, dialysis baths with different concentrations of potassium (2 and 3 meq/l) and calcium (1.25, 1.5 and 1.75 meq/l) were tested on 16 patients. HD bath with less potassium, less calcium and highest bicarbonate concentrations made significant increase in QTc. They found that selection of HD bath must be done on the basis of QT interval before HD (21).

In the cohort study of Buemi et al in 2005, 28 patients underwent dialysis for 4 hours three times per week. First session performed with constant potassium concentration in HD bath and the next two sessions performed with increased and decreased potassium concentrations, respectively. ECGs were done at the start of dialysis (T0), at 15 (T15), 45 (T45), 90 (T90) and 120 minutes (T120) after the beginning of the session, and at the end of treatment (Tend). ECG-derived data (QT, QTd, QTc and QTcd) were calculated and intra- and extracellular potassium concentrated were measured. Highest QT dispersion was seen between 0 to 15 minutes and 15 to 45 minutes and QTc while dialyzing with low potassium HD bath concentration were significantly increased, while intracellular potassium level did not change. They noted that potassium concentration affects the electrical charge of most of the cells (22).

## Objectives

The aim of our study was to investigate the effects of different potassium and bicarbonate concentrations on ECG parameters and the electrochemical balance of cell membranes.

## Patients and Methods

The study is a double blind controlled, crossover trial, on the effect of different dialysate composition on QT interval after HD.

Thirty-five patients older than 16 years who undergoing HD 4 hours three times per week for at least six months entered the study. None of the patients had dysrhythmias, heart failure, active coronary disease and use of antiarrhythmic drugs. HD was performed using a polysulfone membrane, blood flow rate was 300-350 cc/min and dialysate flow was 500-800 cc/min. Dialysate water was purified by reverse osmosis. Each patient underwent a midweek HD session with four different dialysate compositions as follow, in four consecutive weeks. Twelve-lead ECG was recorded by CARWELL electrocardiogram before beginning and one hour after end of dialysis and QT was measured by a cardiologist ( who is uninformed about the HD solutions) in at least three leads and in three consecutive cardiac cycle and the average was recorded. QTc was calculated by Bazett’s formula (QTc=QT/√RR) (11).

A blood sample was taken before HD to evaluate serum calcium (Ca) and bicarbonate.

Our HD solutions include

Solution no.1: K=2 meq/l, HCO3=24 meq/l, Na=140 meq/l, Ca=2.5 meq/l

Solution no.2; K=2 meq/l, HCO3=28 meq/l, Na=140 meq/l, Ca=2.5 meq/l

Solution no.3; K=3 meq/l, HCO3=24 meq/l, Na=140 meq/l, Ca=2.5 meq/l

Solution no.4; K=3 meq/l, HCO3=28 meq/l, Na=140 meq/l, Ca=2.5 meq/l, which were used respectively.

### Ethical issues

1) The research followed the tenets of the Declaration of Helsinki; 2) informed consent was obtained, and they were free to leave the study at any time and 3) the research was approved by the ethical committee of Iran University of Medical Sciences by number; 93/D/105/2128. Registration code in Iran Registry of Clinical Trials is IRCT2013021011194N3 (http://www.irct.ir/).

### Statistical analysis

The results are reported as means ± standard deviation. Student’s *t* test was used. Changes in QTc intervals were evaluated by repeated measures. Correlations were evaluated by univariate regression analysis. The *P* value less than 0.05 was considered statistically significant.

## Results

Mean (±SD) age of patients was 54.38 (±15) years (16 to 77 years), 66.7% were male. The baseline laboratory variables of patients are illustrated in Table 1.

**Table 1:** Demographic and laboratory characters of patients

Number	36
Male (%)	66.7%
Mean age (years)	54.38 ± 15
Serum bicarbonate (meq/L)	21.71 ± 4.74
Serum calcium (meq/L)	8.75 ± 1.04
Dialysis adequacy (KT/V)	1.37 ± 0.45

The changes induced in QTc intervals by different dialysate concentration of K and bicarbonate are shown in Table 2.

**Table 2:** QTC intervals by different composition dialysate

**Dialysate**	**Mean QTC**	**Mean QTC**	**P**
	**Before dialysis**	**After dialysis**	
No 1	434.50 ± 29.64	434.99 ± 43.84	0.946
No 2	428.74 ± 45.71	436.85 ± 32.53	0.226
No 3	440.47 ± 45.71	446.97 ± 34.02	0.239
No 4	437.28 ± 43.84	435.99 ± 83.8	0.771

Dialysate No 1: K=2 meq/L, bicarbonate = 24 meq/L, Ca=2.5 meq/L; Dialysate No 2: K=2 meq/L, bicarbonate=28 meq/L, Ca=2.5 meq/L; Dialysate No 3: K=3 meq/L, bicarbonate=24 meq/L, Ca=2.5 meq/L; Dialysate No 4: K=3 meq/L, bicarbonate=28 meq/L, Ca=2.5 meq/L.

There is no significant change in QTc interval after dialysis in any of dialysis solutions. Longest QTc interval was recorded after dialysis with the third solution (446.97±34.02) and shortest QTc interval was recorded after dialysis with the first solution (434.99±41.76 ms).

The mean differences between QTcs after and before dialysis were 4.96±32.22, 8.1±39.44, 6.5±32.55 and 1.91±39.2, for solutions number 1, 2, 3, 4 respectively, and there was not any significant difference between them.

Long QTc after dialysis (QTc>440 ms) were seen in 61%, 50%, 58% and 38% for dialysate number 1, 2, 3 and 4, respectively.

Comparing mean QTc intervals after dialysis, the only significant difference was seen in comparison of thirdand fourth solution (P=0.03; Table 3).

**Table 3:** Comparison of mean QT intervals after dialysis with four different solutions

**QTc (I)**	**QTc (J)**	**Mean Difference (J)-(I)**	**SE**	**P value**
1	2	-1.863	6.982	0.791
	3	-11.988	6.911	0.092
	4	-0.376	7.779	0.962
2	1	1.863	6.982	0.791
	3	-10.125	5.475	0.073
	4	1.486	6.784	0.828
3	1	11.988	6.911	0.092
	2	10.125	5.475	0.073
	4	11.611	5.114	0.030
4	1	0.376	7.779	0.962
	2	-1.486	6.784	0.828
	3	-11.611	5.114	0.030

Abbreviation: SE, standard error.

Age and gender had not caused any significant correlation with QT intervals before and after dialysis (*P*=0.531 and *P*=0.78, respectively).

Serum bicarbonate level before dialysis had not significant influence on the mean QT interval before and after dialysis (*P*=0.758).

## Discussion

HD appears to cause ECG changes which frequently occur after the start of an HD session, and persist for at least 5 hours after dialysis. These changes appear to have different causes, including dialysis-induced electrolyte alterations (9).

Although the effect of low plasma concentration of potassium and calcium on prolongation of ventricular repolarization has been proven (22), there is controversies about the role of dialysate concentration of potassium (K), calcium (Ca) and bicarbonate on QT interval.

Di Iorio et al studied 22 HD patients with different dialysate bath concentrations of K, Ca and bicarbonate and concluded that the effect of dialysis on QTc appears at the end of dialysis and remains up to 4 hours and mean absolute increase in QTc was observed with dialysate containing Ca**=1.25 meq/l and bicarbonate 34 meq/l whatever the concentration of K was (20).

In the study by Genovesi et al on 16 HD patients, they demonstrated that longest QTcs were seen with low K (2 meq/l) and low Ca (2.5 meq/l) dialysis bath (19). Buemi et al examined two concentrations of K in dialysate, fix K concentration and flexible K with no more than 1 meq/l less than serum K. They concluded that low K gradient between serum and dialysate has a protective role for prolonged repolarization after HD (22).

In our study there is not any significant prolongation of QTc interval after dialysis with different concentrations of K and bicarbonate. It is in contrast to findings of two previous mentioned studies. They noted that the effect of serum K and Ca is more important than dialysate in QTc interval after dialysis (15,23,24).

In single study for evaluation of the effect of dialysate bicarbonate concentration, there was significant difference between bicarbonate 30 and 34 meq/l (22). We evaluated the effect of two different lower concentrations of bicarbonate (24 and 28 meq/l) and it seems that at this level of bicarbonate in dialysate, QTc was not influenced significantly. This low level of bicarbonate in dialysate may have protective role for inducing QT prolongation and arrhythmia after dialysis.

## Conclusion

In conclusion, dialysate with different concentration of K with bicarbonate up to 28 meq/l do not prolong QTc interval after dialysis significantly. Low bicarbonate may have a protective role for QT interval prolongation and arrhythmia after dialysis.

## Limitations of the study

Despite using strict inclusion criteria, the present study has some limitations. The sample size was small and potential confounders such as Ca, K, and bicarbonate after dialysis were not evaluated. However this study had a crossover design and every patient had control of himself or herself, so the small number of patients in the study was not influenced the final results. However, a large study aimed at assessing the impact of dialysate electrolyte and serum electrolyte concentrations before and after dialysis on QTc interval and consequent risk of arrhythmias is essential.

## Authors’ contribution

FS; study design, preparation of manuscript, final revision and data interpretation. SAH; data gathering and manuscript edition. RSJ; data interpretation and manuscript preparation.

## Conflicts of interest

The authors declared no competing interests.

## Ethical considerations

Ethical issues (including plagiarism, data fabrication, double publication) have been completely observed by the authors.

## Funding/Support

This research project was approved by Deputy for Research of Iran University of Medical Science the number of this project is 1390. This project started at September 1, 2014 and finished at February 10, 2015.
